# Alleviation of murine osteoarthritis by deletion of the focal adhesion mechanosensitive adapter, Hic-5

**DOI:** 10.1038/s41598-019-52301-7

**Published:** 2019-10-31

**Authors:** Aya Miyauchi, Joo-ri Kim-Kaneyama, Xiao-Feng Lei, Song Ho Chang, Taku Saito, Shogo Haraguchi, Takuro Miyazaki, Akira Miyazaki

**Affiliations:** 1 Department of Biochemistry, Showa University School of Medicine, Tokyo, Japan; 2 Sensory & Motor System Medicine, Graduate School of Medicine, The University of Tokyo, Tokyo, Japan

**Keywords:** Osteoarthritis, Experimental models of disease

## Abstract

Excessive mechanical stress is a major cause of knee osteoarthritis. However, the mechanism by which the mechanical stress begets osteoarthritis development remains elusive. Hydrogen peroxide-inducible clone-5 (Hic-5; TGFβ1i1), a TGF-β inducible focal adhesion adaptor, has previously been reported as a mediator of mechanotransduction. In this study, we analyzed the *in vivo* function of Hic-5 in development of osteoarthritis, and found that mice lacking Hic-5 showed a significant reduction in development of osteoarthritis in the knee. Furthermore, we found reduced expression of catabolic genes, such as metalloproteinase-13 and a disintegrin and metalloproteinase with thrombospondin type 1 motif 5 in osteoarthritic lesions in mice lacking Hic-5. During osteoarthritis development, Hic-5 is detected in chondrocytes of articular cartilage. To investigate the role of Hic-5 in chondrocytes, we isolated chondrocytes from articular cartilage of wild type and Hic-5-deficient mice. In these primary cultured chondrocytes, Hic-5 deficiency resulted in suppression of catabolic gene expression induced by osteoarthritis-related cytokines such as tumor necrosis factor α and interleukin 1β. Furthermore, Hic-5 deficiency in chondrocytes suppressed catabolic gene expression induced by mechanical stress. Revealing the regulation of chondrocyte catabolism by Hic-5 contributes to understanding the pathophysiology of osteoarthritis induced by mechanical stress.

## Introduction

Osteoarthritis (OA) is the most common joint disease caused by articular cartilage degradation. Some factors such as mechanical stress and inflammatory cytokines induce OA development in association with extracellular matrix (ECM) destruction. The articular cartilage ECM is composed mostly of type II collagen and aggrecan. During early OA development, the expression of the enzymes that degrade cartilage ECM is upregulated. Particularly, matrix metalloproteinase-13 (MMP13) and a disintegrin and metalloproteinase with thrombospondin type 1 motif 5 (ADAMTS5) contribute to type II collagen and aggrecan depletion, respectively. MMP13 is the major type II collagen-degrading protease, and OA progression is inhibited in MMP13-deficient mice through prevention of cartilage erosion, whereas OA-like cartilage erosion is enhanced in MMP13-transgenic mice^1,2^. ADAMTS5 is the main protease responsible for the degradation of aggrecan in mice, and ADAMTS5-defficient mice are protected from experimental OA and inflammatory arthritis^3,4^.

Hydrogen peroxide-inducible clone-5 (Hic-5), isolated as a hydrogen peroxide and transforming growth factor-β1 (TGF-β1) inducible gene, is a LIM-containing adapter protein that mediates cell-matrix adhesion^5^. Hic-5 has emerged as a key regulator of integrin signaling, which leads to actin remodeling, cell migration and proliferation^6^. Genetic deletion of Hic-5 in mice caused no apparent phenotype^7^. However, our recent reports have demonstrated that Hic-5 participates in various pathological processes, such as atherosclerosis^8^, abdominal aortic aneurysm^9^, liver fibrosis^10^ and colorectal carcinogenesis^11^. Previously, we have shown that Hic-5 translocated from focal adhesion to stress fibers in smooth muscle cells undergoing mechanical loading and regulated the contractile capability of the cell^12^. Additionally, Hic-5 plays a role in stretch-induced apoptosis of smooth muscle cells, focal adhesion remodeling in response to mechanical stimuli and arterial remodeling as a mechanosensor^7^. Based on these previous studies, we hypothesized that Hic-5 plays an important role in OA, which is the most common disorder caused by mechanical stress. In the present study, we attempted to clarify the role of Hic-5 in the pathogenesis of OA.

## Results

### Hic-5 deficiency suppresses OA development

**Figure 1:**
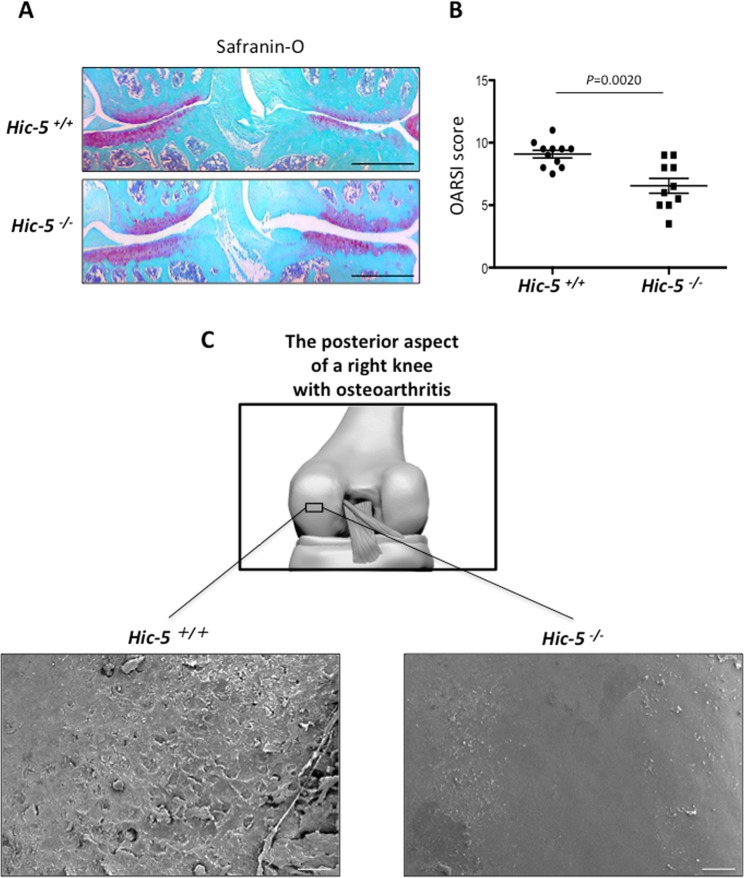
Suppression of OA development in Hic-5^−/−^ mice. (**A**) Safranin-O staining of knee joints 8 weeks after creating a surgical OA model in Hic-5^+/+^ and Hic-5^−/−^ mice. Scale bars, 200 µm. (**B**) OARSI grading of OA development. Data are expressed as means ± SD of Hic-5^+/+^ (n = 11) or Hic-5^−/−^ mice (n = 10); *P < 0.05. (**C**) Scanning electron microscopy images of the posterior aspect of a right knee harvested from Hic-5^+/+^ and Hic-5^−/−^ mice after surgery. Scale bars, 20 µm. The images are representative of three independent experiments.

To determine whether Hic-5 is involved in OA development, we first performed surgical induction of knee OA in Hic-5^+/+^ and Hic-5^−/−^ mice. Safranin-O staining demonstrated that there was significant suppression of cartilage degradation in Hic-5^−/−^ mice compared with Hic-5^+/+^ mice at 8 weeks after surgical induction of OA (Fig. 1A). The Osteoarthritis Research Society International (OARSI) score of Hic-5^−/−^ mice was significantly lower than that of Hic-5^+/+^ mice at 8 weeks after surgery (Fig. 1B). We also observed cartilage degradation in the OA mouse model using SEM, which revealed that cartilage degradation in Hic-5^−/−^ mice was significantly lower that that in Hic-5^+/+^ mice at 8 weeks after surgical induction of OA (Fig. 1C).

### Expression of Hic-5 in articular cartilage and intracellular localization in chondrocytes

**Figure 2:**
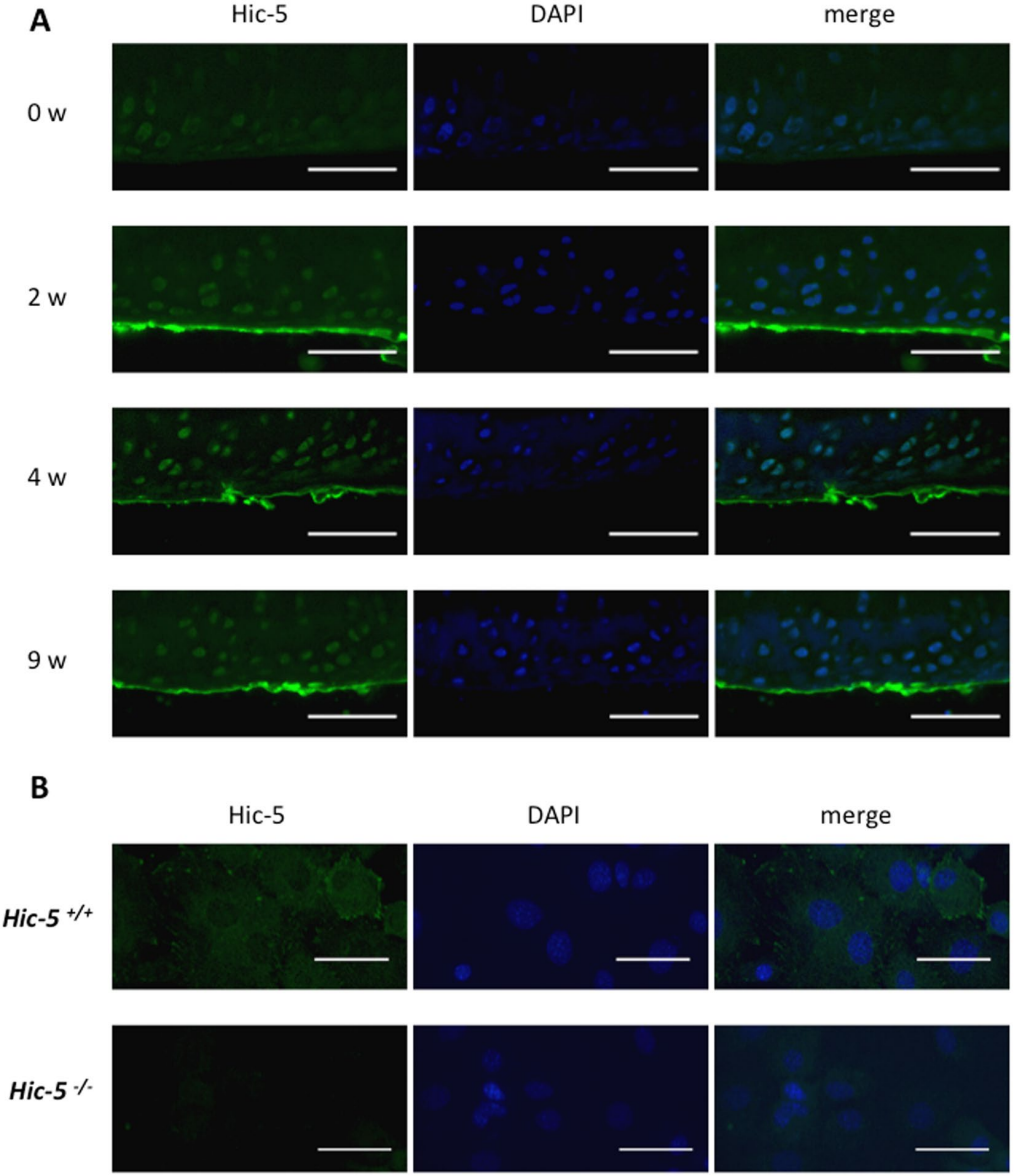
Hic-5 expression during OA development in mouse articular cartilage. (**A**) Sections of uninjured and at 2, 4 and 9 weeks after surgery were collected. Immunohistochemistry was performed with antibodies against Hic-5. Nuclei were stained with DAPI (blue). Scale bars, 100 µm. (**B**) Hic-5 localization in isolated chondrocytes from mouse knee cartilage. Scale bars, 20 µm. One representative result of three independent experiments is shown.

We next examined the expression of Hic-5 in the articular cartilage of a surgically induced knee OA mouse model. Two weeks after surgery, Hic-5 was highly expressed in the superficial layer and in the chondrocytes in the middle transitional zone. This expression pattern was observed continuously from 2 to 9 weeks after surgery (Fig. 2A). Thus, we isolated chondrocytes from mouse knee cartilage and further examined the intracellular localization of Hic-5 in mouse primary chondrocytes (Fig. 2B). Hic-5 was detected at focal adhesions and this localization was similar to other cells expressing Hic-5. Previous studies have demonstrated that Hic-5 serves as a scaffold for interactions of multiple structural and signaling molecules at focal adhesions and that it is involved in ECM organization during mechanical stress, implying that Hic-5 in chondrocytes is involved in OA development as a mediator of mechanical stress^6,12,13^.

### Hic-5 deficiency impairs the expression of catabolic factors in chondrocytes

**Figure 3:**
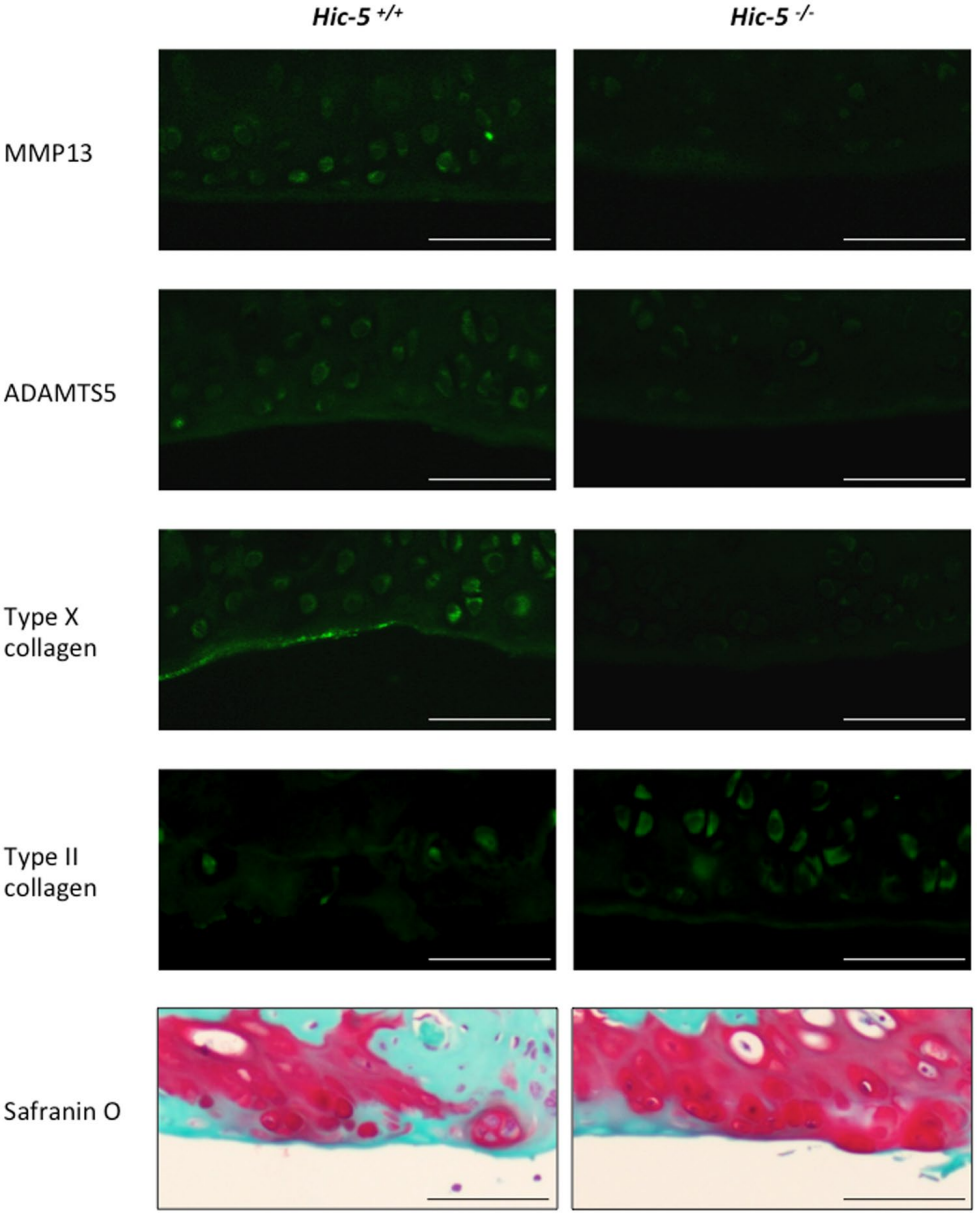
Hic-5 deficiency suppresses the expression of catabolic factors in mouse articular cartilage.Immunofluorescence of MMP13, ADAMTS5, type II collagen and type X collagen in knee cartilage of Hic-5^+/+^ and Hic-5^−/−^ mice at 8 weeks after surgery. Cross-sections were stained with Safranin-O staining (bottom). Scale bars, 100 µm. Data are representative of five independent experiments.

To better understand how Hic-5 is associated with OA development, we next investigated the mechanism of Hic-5 in OA development *in vivo* focusing on ECM remodeling induced by several matrix-degrading enzymes, such as MMP13 and ADAMTS5. Both MMP13 and ADAMTS5 are necessary for cartilage degradation during OA development, and MMP13 degrades type II collagen, which is the main articular component of articular cartilage. Additionally, we examined the expression of type X collagen, which is increased in hypertrophic chondrocytes during OA development. Eight weeks after surgical OA induction, the expression of MMP13, ADAMTS5 and type X collagen was suppressed in Hic-5^−/−^ knee joints, whereas the expression of type II collagen was higher in Hic-5^−/−^ than in Hic-5^+/+^ mice (Fig. 3). These results indicate that the suppression of OA development in Hic-5^−/−^ mice was associated with decreased expression of catabolic factors, such as MMP13 and ADAMTS5.

### Hic-5 enhances the expression of MMP13 and ADAMTS5 induced by inflammatory cytokines or mechanical stress in cultured chondrocytes

**Figure 4:**
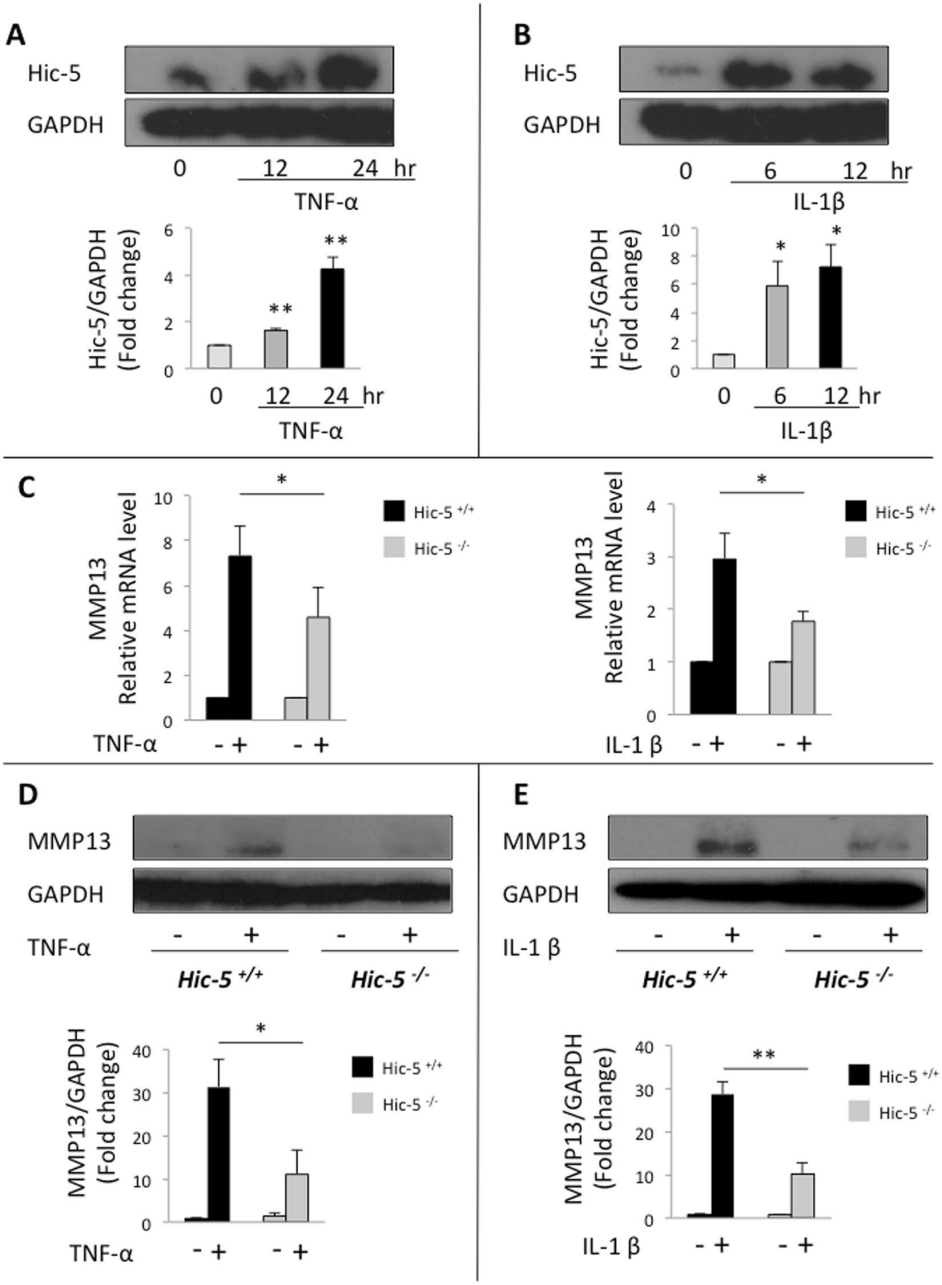
Hic-5 deficiency inhibits inflammatory cytokine-induced MMP13 in chondrocytes. (**A,B**) Hic-5 protein expression in mouse primary chondrocytes stimulated with TNF-α (**A**) or IL-1β (**B**). The lower panels show quantitative analyses of Hic-5 expression after normalization against glyceraldehyde 3-phosphate dehydrogenase (GAPDH). (**C**) mRNA levels of *MMP13* in mouse primary chondrocytes stimulated with IL-1β or TNF-α for 24 h. The mRNA levels were normalized with that of 18 s, and the values relative to the untreated samples are shown. (−), untreated, (+), treated. (**D,E**) Protein expression of MMP13 in mouse primary chondrocytes stimulated with IL-1β or TNF-α for 24 h. The lower panels show quantitative analyses of MMP13 expression after normalization against GAPDH. (−), untreated, (+), treated. The images are representative of three independent experiments. All data are expressed as means ± SD. *P < 0.05, **P < 0.01.

To reveal the catabolic effects caused by Hic-5, we first treated mouse primary chondrocytes with tumor necrosis factor α (TNF-α) or interleukin-1β (IL-1β), which are inflammatory cytokines implicated in OA pathogenesis. Western blot analysis showed that Hic-5 was induced by both TNF-α and IL-1β (Fig. 4A,B). Then, we compared the expression of MMP13 in Hic-5^−/−^ and Hic-5^+/+^ chondrocytes. After treatment of mouse primary chondrocytes with TNF-α or IL-1β, we found a significant reduction in *MMP13* mRNA level in Hic-5^−/−^, compared with Hic-5^+/+^ chondrocytes (Fig. 4C). In addition, MMP13 protein expression levels were suppressed in Hic-5^−/−^ chondrocytes compared with Hic-5^+/+^ (Fig. 4D,E). These data demonstrated that the expression of MMP13 was mediated by Hic-5 in mouse primary chondrocytes, which is consistent with the OA tissue in Hic-5^+/+^ and Hic-5^−/−^ mice.

**Figure 5:**
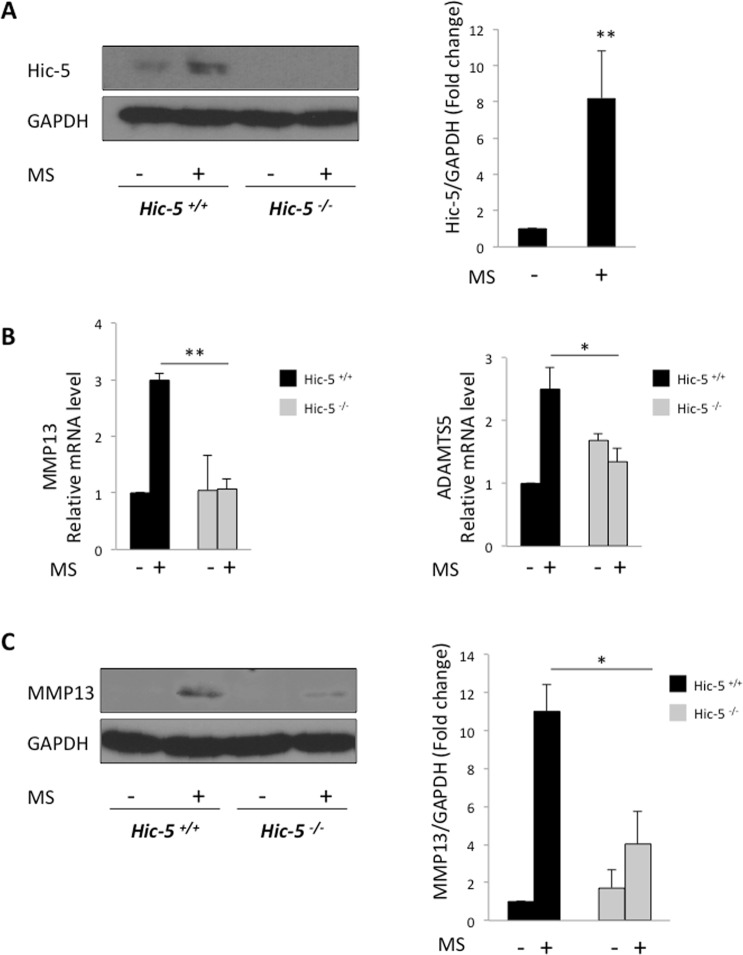
The expression of MMP13 and ADAMTS5 induced by mechanical stretch was suppressed in Hic-5^−/−^ chondrocytes. (**A**) Hic-5 protein expression in mouse primary chondrocytes after tensile stress loading (+), or without loading (−). Cells were cultured for up to 12 h after uniaxial cyclic tensile strain (10%, 0.5 Hz, 30 min). The right panel shows quantitative analysis of Hic-5 expression after normalization against GAPDH. MS: mechanical stress. (**B**) mRNA levels of *MMP13* and *ADAMTS5* in mouse primary chondrocytes after tensile stress loading (+), or without loading (−). (**C**) MMP13 protein expression in mouse primary chondrocytes after tensile strain loading (+), or without loading (−). The right panel shows quantitative analysis of MMP13 expression after normalization against GAPDH. Representative data of five (**A**) or three (**B,C**) independent experiments are shown and expressed as means ± SD. *P < 0.05, **P < 0.01.

A previous *in vitro* study using the cell-stretcher system demonstrated that mechanical loading induced the expression MMP13 and ADAMTS5^14^. Furthermore, we have previously shown that Hic-5 regulated vascular remodeling through mechanical stress^7^. Thus, we first examined the expression of Hic-5 in mouse primary chondrocytes after a 0.5-Hz, 10% cyclic strain loading for 30 min. Hic-5 protein expression markedly increased (Fig. 5A). To confirm whether Hic-5 is required for the expression of MMP13 and ADAMTS5 induced by mechanical loading, we performed real-time RT-qPCR on chondrocyte mRNA samples 12 h after mechanical loading. Hic-5^+/+^ chondrocytes showed high mRNA levels of *mmp13* and *adamts5* after mechanical loading, however in Hic-5^−/−^ chondrocytes they were significantly reduced (Fig. 5B). Furthermore, western blot analysis indicated that MMP13 protein levels in Hic-5^−/−^ chondrocytes were suppressed after mechanical loading (Fig. 5C). These results suggest that Hic-5 contributes to OA development by regulating the expression of MMP13 and ADAMTS5 induced by inflammatory cytokines or mechanical loading.

## Discussion

In this study, we report that Hic-5 plays a key role as a mediator of mechanical force in the development of OA. Genetic deletion of *hic-5* in mice resulted in suppression of OA development through inhibition of catabolic genes’ expression such as MMP13 and ADAMTS5, the major catabolic molecules responsible for OA lesion formation. Furthermore, Hic-5-deficient chondrocytes showed suppressed expression of these catabolic factors induced by inflammatory cytokines or mechanical loading. These data indicate that Hic-5 exerts catabolic effects in articular cartilage degeneration.

Hic-5 is a non-enzymatic adaptor protein coordinating multiple protein-protein interactions by acting as a scaffold both in focal adhesions and in the nucleus. The types of molecules for which Hic-5 acts as a scaffold differ depending on the cell type and tissue environment. For example, we have reported that Hic-5 acts as a scaffold for the MKK4/p54 JNK pathway to induce MT1-MMP in the development of abdominal aortic aneurysms^9^. Moreover, Hic-5 functions as a scaffold for the TGF-β/Smad2 pathway and its deficiency significantly attenuated mouse liver fibrosis because of reduced collagen production^10,15^. Hic-5 also functions as a scaffold for stabilizing focal adhesion components in the mechanosensitive remodeling processes of vascular walls^7^. These results raise the possibility that Hic-5 mediates the required intermolecular reactions to create a stimuli-responsive extracellular microenvironment in various disorders, and eventually regulates ECM remodeling in a context-specific manner. In OA cartilage, Hic-5 induced the expression of MMP13 and ADAMTS5, which severely affected ECM remodeling during OA development. It is generally assumed that ADAMTS5 and ADAMTS4 are the aggrecanases leading to OA. However, previous studies have found a significant reduction in the severity of cartilage destruction in ADAMTS5^−/−^ mice, whereas there was no effect in ADAMTS4^−/−^ mice^3,16^. Therefore, we consider that ADAMTS5, rather than ADAMTS4, is responsible for disease progression in a surgical OA model in mice, and thus we only analyzed ADAMTS5 in this study.

Furthermore, we observed that Hic-5 deficiency actually suppressed the onset of OA *in vivo*. While the lack of sham controls is a limitation of this study, the suppression of OA development correlated with decreased expression of catabolic factors in Hic-5^−/−^ mice. Therefore, Hic-5 seems to be a crucial regulator of ECM remodeling in cartilage by inducing the expression of ECM-degrading enzymes and Col10a.

The effect of mechanical stress on OA pathology is well recognized, and recent studies have revealed that the cytoskeletal architecture, called primary cilium, is related to mechanotransduction^17,18^. Primary cilia are microtubule-based cell surface protrusions that function as sensors of mechanical and chemical environmental cues. Molecules known as mechanotransducers in other cell types including integrins are present on chondrocyte cilia, and chondrocytes lacking cilia fail to respond to mechanical force. Chang *et al*. have reported that depletion of primary cilia in articular chondrocytes resulted in increased expression of OA markers including MMP13, ADAMTS5, ColX and Runx2^19^. We have previously shown that Hic-5 was involved in the formation of microvilli-like structures, which are strikingly similar to primary cilia, on the endothelial cell surface in atherosclerosis^8^. Interestingly, a recent study has revealed that primary cilia have the capacity of actin polymerization. Indeed, F-actin localized in primary cilia, and intraciliary actin polymerization is essential for cilia function^20^. Furthermore, several groups have shown that Hic-5 serves as a mechanosensor and regulator of the actin cytoskeleton and focal adhesions^7,21–24^. Taken together, these findings imply that Hic-5 may coordinate its function as a mechanosensor with primary cilia by regulating actin cytoskeleton in articular chondrocytes. Further analysis is required to assess how Hic-5 translates mechanical force into pathogenic signals and regulates catabolic factors’ expression in cartilage.

In conclusion, we found that Hic-5 is a mediator of mechanical force-induced lesion formation in OA. Our findings may help understand the mechanical force-induced signaling, which may become a therapeutic target for OA.

## Materials and Methods

### Animals

All experiments were performed with protocols approved by the regional Animal Study Committees of Showa University School of Medicine. And we confirmed that all methods were carried out in accordance with relevant guidelines and regulations. Wild type (Hic-5^+/+^) and systemic Hic-5-knockout (Hic-5^−/−^) mice (C57BL/6 background) were housed in a temperature and humidity controlled environment.

### Cell cultures

Primary articular chondrocytes were isolated from 5-day-old Hic-5^+/+^ and Hic-5^−/−^ mice, as described previously^25^. Briefly, we isolated the femoral head, femoral condyle, and tibial plateau using a scalpel. These pieces of cartilage were incubated in collagenase D solution. The next day, we retrieved the collagenase D solution with residual cartilage and successively passed the solution through pipettes to disperse any cell aggregates. The cell suspension of isolated cells was filtered through a strainer and then seeded on a culture dish. Primary chondrocytes were maintained in Dulbecco’s Modified Eagle’s Media (DMEM; Wako, Osaka, Japan) with low glucose (1 g/l) containing 10% fetal bovine serum (Life technologies, CA, USA). For most experiments, primary cells were transferred to serum-free DMEM for 24 h before being exposed to stimuli.

### OA experiments

The surgical procedure for establishing an experimental OA model was performed as previously described using 8-week-old mice^26^. Under general anesthesia, we performed resection of the medial collateral ligament and the medial meniscus under a surgical microscope. After surgery, all mice were maintained under the same conditions and were analyzed 8 weeks later. We quantified OA severity using the OARSI system^27^. Briefly, an observer (blinded) assigned a score between 0 and 6 in all four quadrants and the severity of OA was expressed as the summed scores. These scores were combined for the areas of the medial tibial plateau, medial femoral condyle, lateral tibial plateau, and lateral femoral condyle.

### Scanning electron microscopy

The samples were processed using procedures described previously^8^. Briefly, articular cartilages were harvested from the mice 8 weeks after surgery, followed by immersion fixation with 2.5% glutaraldehyde for 24 h at 4 °C. The specimens were washed with phosphate-buffered saline (PBS) and stained with 1% buffered osmium tetroxide. Then, they were dehydrated by passing through an ethanol gradient, freeze-dried, coated with platinum and observed with an S-4700 scanning electron microscope (SEM; Hitachi, Tokyo, Japan) at 25 kV.

### Histological analyses

Tissue samples were fixed in 4% paraformaldehyde buffered with PBS at 4 °C for 1 day, decalcified with 10% EDTA (pH 7.4) at 4 °C for 2 weeks and embedded in paraffin. Sagittal sections (4-μm thick) were cut from specimens. For immunofluorescence, sections were incubated with antibodies against ADAMTS5 (1:50; D-16, Santa Cruz Biotechnology, TX, USA), type II collagen (1:200; LB-1297, LSL, Tokyo, Japan), type X collagen (1:200; LB-0092, LSL, Tokyo, Japan), Hic-5 (1:200; 611165, BD Biosciences, NJ, USA) and MMP13 (1:100; 18165–1-AP, Proteintech, IL, USA) diluted in the blocking reagent. For the staining of ADAMTS5, MMP13, type II collagen and type X collagen we used the CSA II Biotin-Free Catalyzed Amplification System (Agilent Technologies, CA, USA) and DAPI. Hic-5 staining in chondrocytes was performed by the same procedure as immunofluorescence. At least three samples were tested in each assay.

### Western blotting

Chondrocyte lysates were separated by SDS-PAGE, transferred to polyvinylidene difluoride membranes (GE Healthcare, MA, USA) and blocked with 0.5% BSA in PBS containing 0.1% Tween 20. Membranes were blotted with primary antibodies against Hic-5, GAPDH (1:2000; 171–3, MBL, Nagoya, Japan) and MMP13 (1:400; MAB13426, Millipore, MA, USA). After incubation with horseradish peroxidase-conjugated secondary antibodies, positive bands were visualized using Western Lightning chemiluminescence reagent (Wako, Osaka, Japan), followed by exposure to X-ray film (Fuji Film, Tokyo, Japan). The band densities were measured using the Densitograph software (ATTO, Tokyo, Japan).

### Real-time qRT-PCR

Real-time PCR was performed to quantify chondrocyte gene expression using the RT-qPCR kit (TaKaRa, Shiga, Japan) and an ABI7900 real-time PCR detection system (Applied Bio systems, CA, USA). The expression values were normalized against a housekeeping gene (*18* *s*), and fold changes were calculated relative to the control group using the 2^−∆∆^ Ct method.

Primers used: *18s*-forward (F): 5′-TAGAGGGACAAGTGGCGTTC-3′, reverse (R): 5′-CGCTGAGGCCAGTCAGTGT-3′; *adamts5*-F: 5′-GCCATTGTAATAACCCTGCACC-3′, R: 5′-TCAGTCCCATCCGTAACCTTTG-3′; *mmp13*-F: 5′-TGATGGACCTTCTGGTCTTCTGG-3′, R: 5′-CATCCACATGGTTGGGAAGTTCT-3′.

### Cyclic tensile strain loading of mouse primary chondrocytes

We seeded mouse primary chondrocytes on silicon stretch chambers coated with collagen at a density of 2.5 × 10^4^ cells/chamber. Each chamber had a culture surface of 2 × 2 cm. After culturing for 48 h, cyclic tensile strain (0.5 Hz, 10% elongation) was applied for 30 min using a uniaxial stretching system (NS-550; STREX, Osaka, Japan) at 37 °C and 5% CO_2_. We seeded control cells on the same chambers and cultured them without cyclic tensile strain. Primary chondrocytes were collected 12 h after stretching.

### Statistical analyses

All data are expressed as the mean ± SD of three or five independent samples, with more than three samples per group. For comparisons between two groups, the Student’s unpaired two-tailed *t*-test was applied. *P* < 0.05 was considered significant.

## Supplementary information

Supplementary Dataset 6
