# Supplementary material for: Alleviation of murine osteoarthritis by deletion of the focal adhesion mechanosensitive adapter, Hic-5
Source: Sci Rep. 2019 Oct 31;9:15770. doi: 10.1038/s41598-019-52301-7 (PMC6823501; doi:10.1038/s41598-019-52301-7)
Supplement: Supplementary file 1 — Supplementary Dataset 6 [file 41598_2019_52301_MOESM1_ESM.pdf]

SUPPLEMENTAL MATERIAL

Alleviation of murine osteoarthritis by deletion of the focal adhesion mechanosensitive adapter, Hic-5

Aya Miyauchi[1], Joo-ri Kim-Kaneyama[1*], Xiao-Feng Lei[1], Song Ho Chang[2], Taku Saito[2], Shogo Haraguchi[1], Takuro Miyazaki[1] and Akira Miyazaki[1]

[1]Department of Biochemistry, Showa University School of Medicine, Tokyo, Japan
[2]Sensory & Motor System Medicine, Graduate School of Medicine, The University of Tokyo, Tokyo, Japan

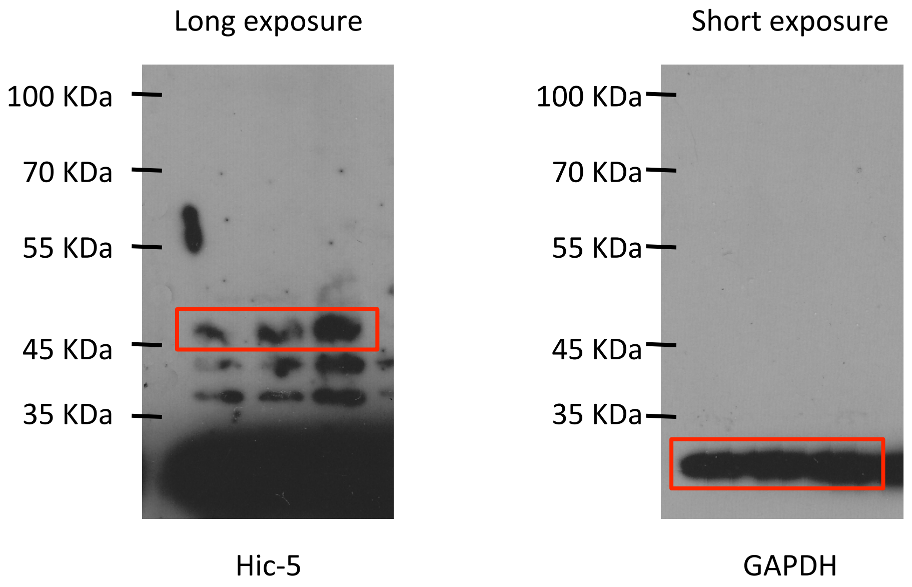

Supplementary Figure S1. Uncropped scans of Western blots. (Fig. 4A)

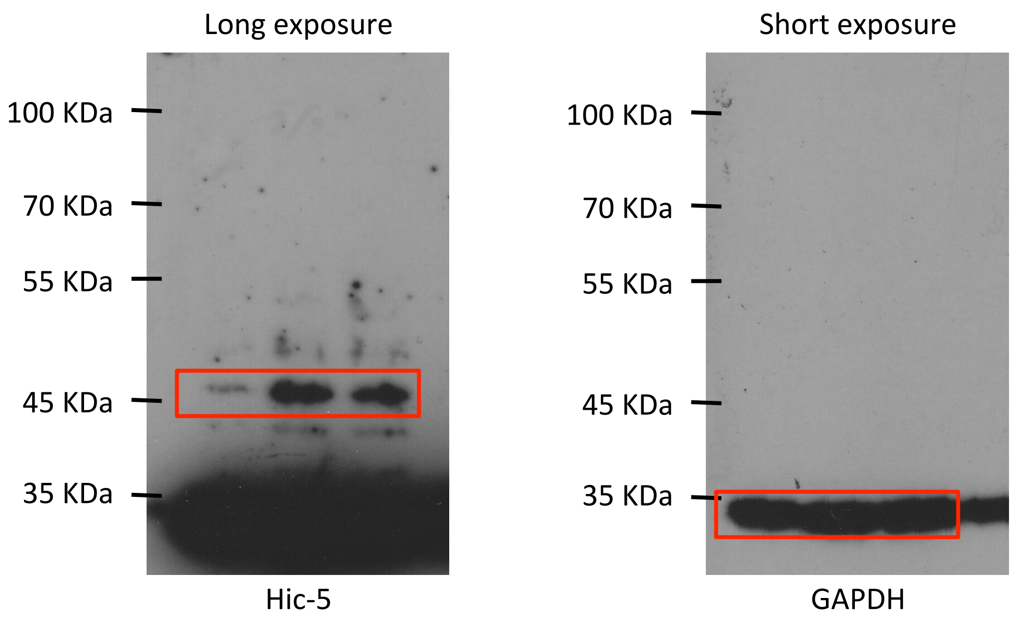

Supplementary Figure S2. Uncropped scans of Western blots. (Fig. 4B)

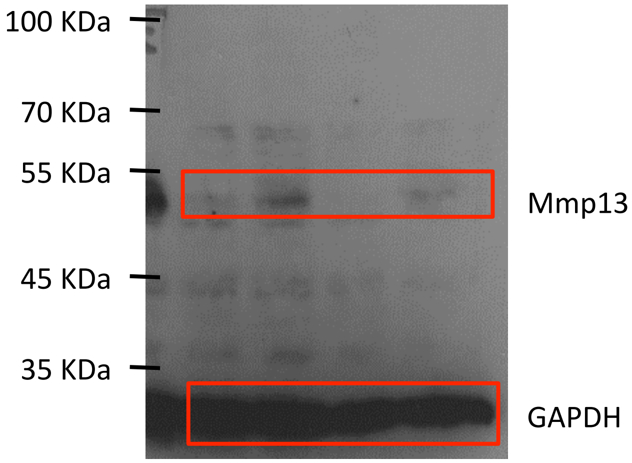

Supplementary Figure S3. Uncropped scans of Western blots. (Fig. 4D)

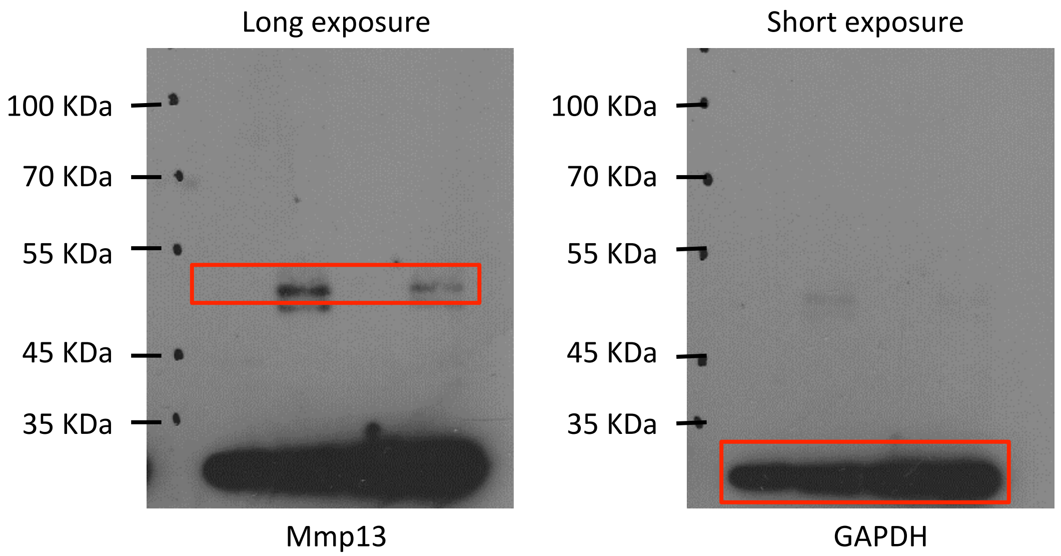

Supplementary Figure S4. Uncropped scans of Western blots. (Fig. 4E)

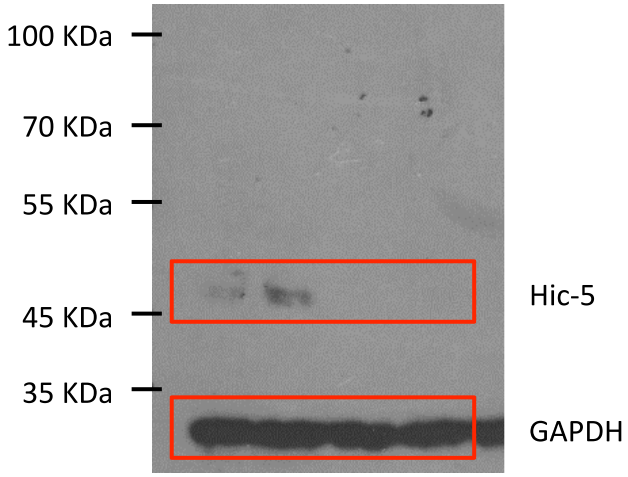

Supplementary Figure S5. Uncropped scans of Western blots. (Fig. 5A)

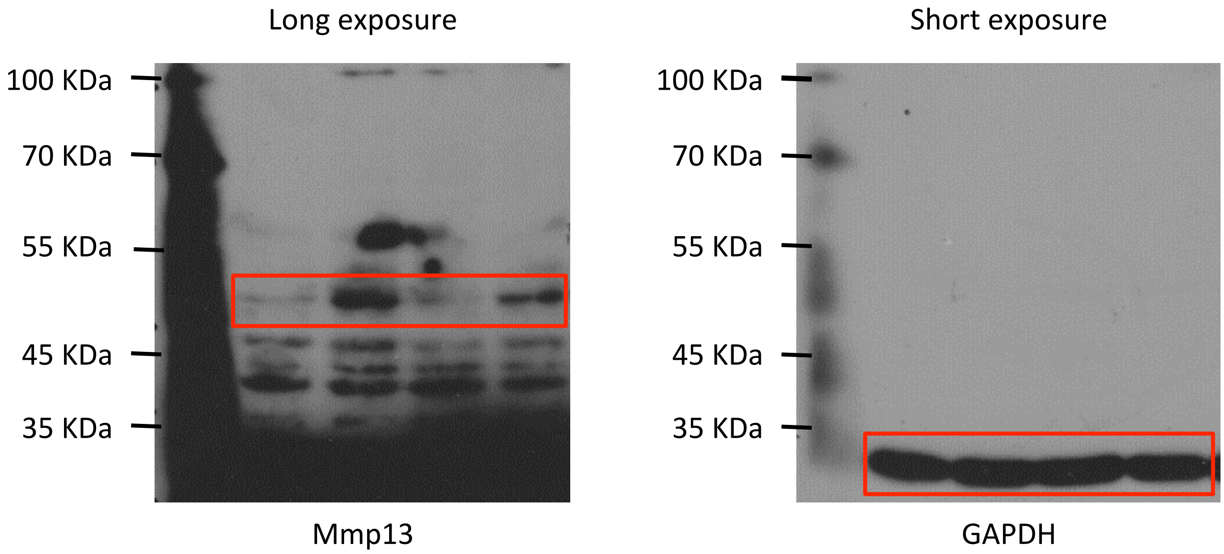

Supplementary Figure S6. Uncropped scans of Western blots. (Fig. 5C)
